# A novel gene-trap line reveals the dynamic patterns and essential roles of *cysteine and glycine-rich protein 3* in zebrafish heart development and regeneration

**DOI:** 10.1007/s00018-024-05189-0

**Published:** 2024-03-31

**Authors:** Shuzhang Liang, Yating Zhou, Yue Chang, Jiayi Li, Min Zhang, Peng Gao, Qi Li, Hong Yu, Koichi Kawakami, Jinmin Ma, Ruilin Zhang

**Affiliations:** 1 TaiKang Medical School (School of Basic Medical Sciences), Wuhan University, Wuhan, 430071 China; 2 School of Life Sciences, Fudan University, Shanghai, 200433 China; 3 Shanghai Key Laboratory of Regulatory Biology, Institute of Molecular Medicine, School of Life Sciences, East China Normal University, Shanghai, 200241 China; 4 Shanghai Pediatric Congenital Heart Disease Institute and Pediatric Translational Medicine Institute, Shanghai Children’s Medical Center, Shanghai Jiao Tong University School of Medicine, Shanghai, 200127 China; 5 Institute of Myocardial Injury and Repair, Wuhan University, Wuhan, 430071 China; 6 Hubei Provincial Key Laboratory of Developmentally Originated Disease, Wuhan, 430071 China; 7 Laboratory of Molecular and Developmental Biology, National Institute of Genetics, Mishima, Shizuoka 411-8540 Japan; 8 Department of Genetics, Graduate University for Advanced Studies (SOKENDAI), Mishima, Shizuoka 411-8540 Japan; 9 Medical Frontier Innovation Research Center, The First Hospital of Lanzhou University, The First Clinical Medical College of Lanzhou University, Lanzhou, 730000 China

**Keywords:** Gene-trap, *csrp3/mlp*, Heart development, Trabeculation, Heart regeneration

## Abstract

Mutations in cysteine and glycine-rich protein 3 (CSRP3)/muscle LIM protein (MLP), a key regulator of striated muscle function, have been linked to hypertrophic cardiomyopathy (HCM) and dilated cardiomyopathy (DCM) in patients. However, the roles of CSRP3 in heart development and regeneration are not completely understood. In this study, we characterized a novel zebrafish gene-trap line, *gSAIzGFFM218A*, which harbors an insertion in the *csrp3* genomic locus, heterozygous fish served as a *csrp3* expression reporter line and homozygous fish served as a *csrp3* mutant line. We discovered that *csrp3* is specifically expressed in larval ventricular cardiomyocytes (CMs) and that *csrp3* deficiency leads to excessive trabeculation, a common feature of CSRP3-related HCM and DCM. We further revealed that *csrp3* expression increased in response to different cardiac injuries and was regulated by several signaling pathways vital for heart regeneration. Csrp3 deficiency impeded zebrafish heart regeneration by impairing CM dedifferentiation, hindering sarcomere reassembly, and reducing CM proliferation while aggravating apoptosis. Csrp3 overexpression promoted CM proliferation after injury and ameliorated the impairment of ventricle regeneration caused by pharmacological inhibition of multiple signaling pathways. Our study highlights the critical role of Csrp3 in both zebrafish heart development and regeneration, and provides a valuable animal model for further functional exploration that will shed light on the molecular pathogenesis of CSRP3-related human cardiac diseases.

**Supplementary Information:**

The online version contains supplementary material available at 10.1007/s00018-024-05189-0.

## Introduction

Cysteine and glycine-rich protein 3 (CSRP3), also known as muscle LIM protein (MLP), expresses in both the myocardium and skeletal muscles and serves as a scaffold protein and a transcription cofactor important for the maintenance of normal muscle structure and function [1]. Evidence has highlighted the role of CSRP3 as a mechanosensor capable of converting extracellular mechanical stimuli into intracellular biochemical signals, and triggering downstream signals for myocyte growth and survival [2, 3]. Mutations in the *CSRP3* gene have been linked to dilated cardiomyopathies (DCM) and hypertrophic cardiomyopathies (HCM) [4–6]. While considerable efforts have been undertaken to understand the underlying mechanisms, the role of CSRP3 in heart development and regeneration as well as in the etiology of relevant diseases still warrants further investigation.

Zebrafish are excellent model animals for studies of organ development and human diseases, and novel genetic tools are continuously being developed. Taking advantage of *Tol2* transposable elements and Gal4-UAS systems, various gene-trap lines have been generated in which Gal4 is inserted into proper intron regions in the genome mediated by *Tol2* transposase [7, 8]. When gene-trap lines were crossed with transgenic fish carrying fluorescent reporter genes downstream of UAS, the expression of the targeted gene can be visualized. Heterozygous fish can be used as reliable reporter lines for endogenous gene expression, whereas homozygous fish represent mutants of the targeted genes. Collections of gene-trap lines have proven to be valuable resources for studying gene function [9, 10].

In the present study, we characterized a novel zebrafish gene-trap line, *gSAIzGFFM218A*, which harbors an insertion in the *csrp3* genomic locus and we further explored the role of Csrp3 in heart development and regeneration. We revealed that Csrp3 deficiency in zebrafish led to excessive trabeculation and impeded zebrafish ventricle regeneration. *csrp3* expression was dynamic during heart development and regeneration, and was influenced by hemodynamic alteration, Notch signaling, and other pathways vital for heart regeneration. Csrp3 overexpression in zebrafish promoted CM proliferation after injury and heart regeneration. Our study highlights the critical role of Csrp3 in zebrafish heart development and regeneration, and provides a valuable animal model for further functional exploration which will facilitate the understanding of the molecular pathogenesis of CSRP3-related human cardiac diseases.

## Materials and methods

### Zebrafish husbandry

Zebrafish were raised and maintained under standard conditions. Zebrafish used in this study included wild-type AB strain, transgenic lines *Tg(cmlc2:nDsRed), Tg(cmlc2:mCherry)*, *Tg(flk:mCherry), Tg(vmhc:mCherry-NTR)*, *Tg(tp1:d2GFP)*, *Tg(cmlc2:Csrp3-EGFP)*, and gene-trap line *Tg(gSAIzGFFM218A;UAS:GFP)*. To prevent pigmentation, embryos were incubated with 0.003% PTU (1-phenyl-2-thiourea) in E3 water from 24 hpf. All experiments were performed in accordance with institutional and national animal welfare guidelines.

### Chemical treatment

To perform ventricular CM ablation, *Tg(vmhc:mCherry-NTR)* larvae were treated with 6 mM MTZ (metronidazole, Sigma-Aldrich) in E3 water for 4 h at 3 dpf as previously described [11]. To modulate signaling pathways, larvae were incubated with the following chemicals for the indicated time period: 100 μM DAPT (Sigma-Aldrich), 12 μM AG1478 (Sigma-Aldrich), 5 μM cardiomogen-1 (Sigma-Aldrich), 7.5 μM dorsomorphin (Sigma-Aldrich), 5 μM LDN193189 (Selleck), or 10 μM rapamycin (Cell Signaling Technology). To stop blood flow, larvae were treated with 1.8 mM tricaine (3-aminobenzoic acid ethyl ester, Sigma-Aldrich) or 10 mM BDM (2,3-butanedione monoxime, Sigma-Aldrich) in E3 water for the indicated time period, and then washed with fresh E3 water.

### Cryoinjury of adult zebrafish heart

The ventricular cryoinjury was performed according to a previously established procedure [12]. Briefly, adult zebrafish at 6–12 months of age were anesthetized and placed ventral side up in a moistened sponge. Pericardial sac was exposed by removing surface scales and a small piece of skin with an incision. The apex of the ventricle was gently pulled up and further frozen with a precooled cryoprobe for 5 s. The fish were then placed back into a water tank, and water was puffed over the gills with a plastic pipette until they breathed and swam regularly.

### Morpholino injection

Morpholino injections were performed as previously described [13]. The morpholino against *tnnt2a* (5′-CATGTTTGCTCTGATCTGACACGCA-3′) was purchased from Gene-tools, dissolved in nuclease-free water containing 10% phenol red, then injected into one-cell stage embryos. All injected embryos and larvae were used for relevant experiments at indicated stages.

### Generation of *Tg(cmlc2:Csrp3-EGFP)* zebrafish

To generate *Tg(cmlc2:Csrp3-EGFP)* zebrafish, HiFi DNA Assembly kit (NEB) was used to simultaneously clone *cmlc2* promoter, full length *csrp3* CDS (without a stop codon) and EGFP fragments into pDESTol2pA2 destination vector. After sequence verification, DNA construct was co-injected with Tol2 transposase mRNA into embryos at the one-cell stage. Injected embryos were raised to adult, positive founders were outcrossed with wild-type fish to generate the stable transgenic line.

### Immunofluorescence

For PCNA staining, adult zebrafish hearts were extracted and fixed in 4% paraformaldehyde for 1 h at room temperature, and subsequently dehydrated in 30% sucrose, then embedded in OCT solution and further sectioned at 10 μm thickness with a Leica cryostat. Immunostaining on cryosections was performed as previously described [14], administrated with antigen retrieval at 98 °C for 20 min in the citric acid buffer. CM proliferation index was calculated as the number of PCNA^+^Mef2C^+^ cells over the number of total Mef2C^+^ cells. Whole-mount larvae immunofluorescence was performed as previously described [15]. The primary and secondary antibodies used in this study were listed in Supplementary Table 2. Images were obtained using a Leica SP8 confocal microscope.

### Histochemical staining

Injured and control zebrafish hearts were extracted, fixed, dehydrated, and embedded in paraffin and then sectioned at 5 μm thickness. Histochemical staining was performed on paraffin-embedded heart sections according to the manufacturer protocol of hematoxylin–eosin staining kit (Beyotime Biotech). Acid Fuchsin Orange-G (AFOG) staining for fibrin and fibrotic scar analyses was performed on 10 μm cryosections at 30 dpa as previously described [16]. Images were taken using an Olympus IX83 Microscope.

### Western blotting

Larvae were homogenized in cold RIPA lysis buffer supplemented with complete proteinase inhibitors. Protein lysates were separated on 12% SDS-PAGE and transferred to PVDF membranes according to standard protocol [15]. The membranes were then incubated with anti-Csrp3 or anti-α-tubulin primary antibody, and further with HRP-conjugated anti-rabbit IgG or anti-mouse IgG secondary antibody (CWBio). Signals were visualized with Clarity Western ECL Substrate using a ChemiScope series system (Clinx).

### in situ hybridization

Whole-mount in situ hybridization (WISH) was performed as previously described [11], using the following probes: *csrp3*, *nkx2.5*, *hand2*, *tp53*, *fosl2*, *piezo1*, *ilk*. Antisense riboprobes were synthesized in vitro using T7 RNA polymerase (NEB) with DIG RNA Labeling Mix. Transcription templates were amplified from cDNA using specific primers listed in Supplementary Table 1. The signal was detected using anti-digoxigenin-AP (Roche) and visualized by NBT/BCIP substrate (Roche). Images were taken by a Nikon SMZ18 stereo microscope. Fluorescence in situ hybridization (FISH) of *csrp3* expression on cryosections was performed as previously described [17], signals were detected using anti-digoxigenin-POD (Roche) and visualized by TSA plus fluorescence kit (PerkinElmer), subsequently conducted immunofluorescence with Tnnt2 antibody as described above. FISH images were obtained using a Leica SP8 confocal microscope.

### Quantification and statistical analysis

ImageJ software was used to measure the size of the scar area. Statistical tests and p-values are indicated in the figure legends. All statistical values were presented as mean ± SD and the statistical significance was defined as *P < 0.05, **P < 0.01, ***P < 0.001 and ****P < 0.0001 determined by Student’s t-test or the chi-squared test using SPSS and GraphPad Prism 9.0 software.

## Results

### *gSAIzGFFM218A *directs GFP expression in zebrafish hearts and harbors an insertion in the *csrp3* genomic locus

**Fig. 1:**
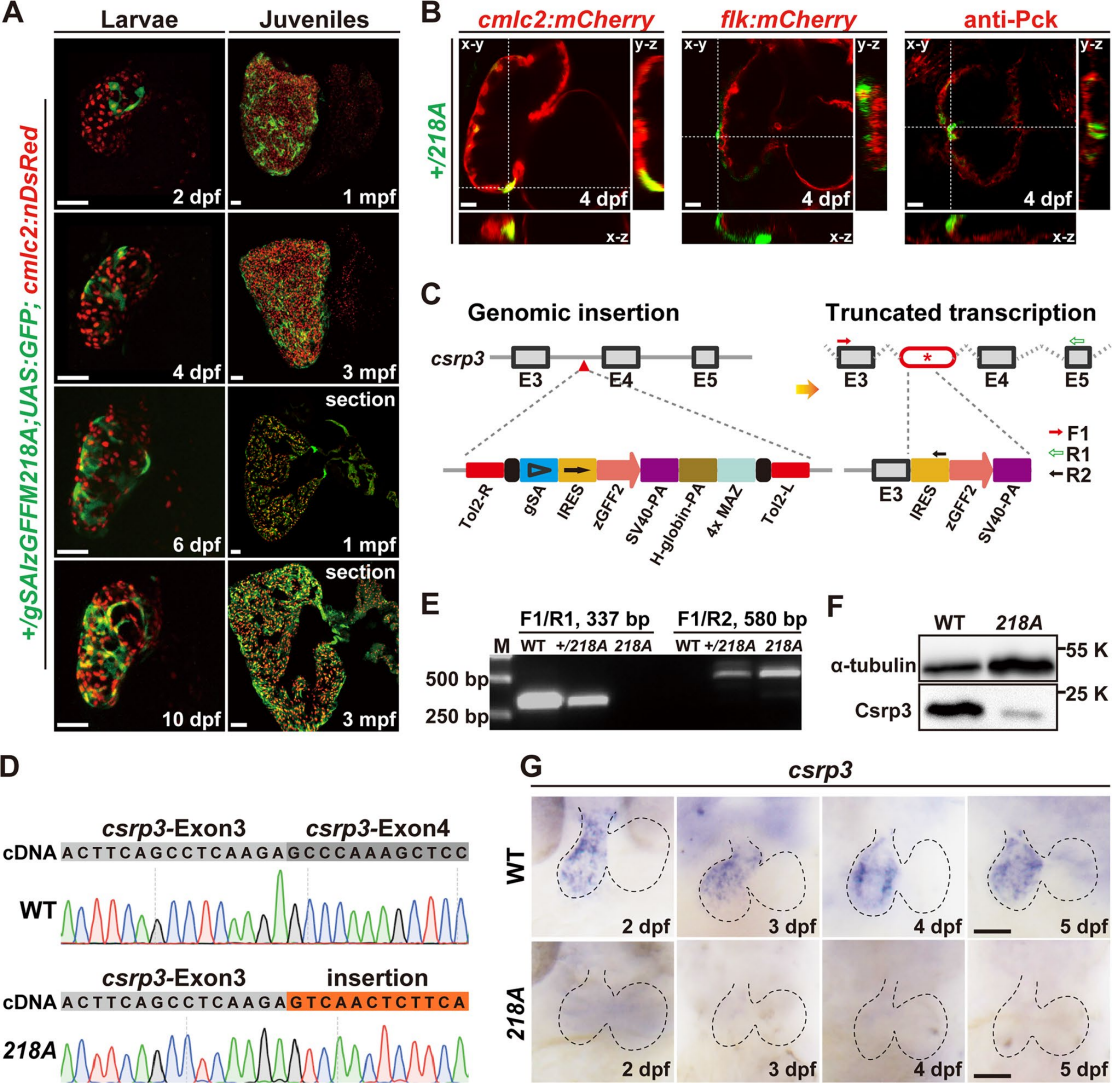
*gSAIzGFFM218A* directs GFP expression in zebrafish hearts and harbors an insertion in the *csrp3* genomic locus.** A** Mosaic GFP expression in the hearts of heterozygous *gSAIzGFFM218A* (+ */218A*) larvae and juveniles at indicated stages. Scale bars, 50 μm. **B** GFP in + */218A* overlapped with the myocardial marker (*cmlc2:mCherry* reporter), but neither with the endocardial marker (*flk:mCherry* reporter) nor the epicardial marker (anti-Pck staining). Scale bars, 20 μm. **C** Schematic diagram illustrated the components of the gene-trap construct and its insertion in intron 3 of the *csrp3* locus, which was predicted to result in a truncated *csrp3* transcription. **D** Sequencing analysis of *csrp3* cDNA from wild-type or homozygous *218A* larvae. **E** RT-PCR analysis of *csrp3* transcripts from wild-type, + */218A,* and *218A* larvae using specific primers indicated in **C**. M, molecular weight markers. **F** Western blotting revealed a dramatic reduction in Csrp3 protein level in *218A* mutants. **G** Whole-mount in situ hybridization (WISH) showed the endogenous *csrp3* expression in wild-type or *218A* larval hearts. Scale bars, 50 μm

*gSAIzGFFM218A* was generated through the *Tol2* transposon-mediated gene-trap approach described previously that drives the expression of Gal4FF, an engineered Gal4 transcription activator in specific cell types [7, 18]. To visualize the expression of Gal4FF, *gSAIzGFFM218A* was mated with *Tg(UAS:GFP)* to obtain the double transgenic line *Tg(gSAIzGFFM218A;UAS:GFP)*, referred to as *218A* hereafter. Heterozygous *218A* (+ */218A*) fish exhibited GFP expression in the heart and gut and faintly in the somites (Figure S1A). Interestingly, the fluorescence pattern in the heart was restricted to the ventricle during early larval development and was noted as early as 2 days post-fertilization (dpf). By 10 dpf, the fluorescence displayed a dynamic mosaic pattern, gradually increased over time, and became widespread throughout the adult ventricle at 3 months post-fertilization (mpf), with scattered fluorescence seen in the adult atrium occasionally (Fig. 1A). To precisely determine the localization of GFP fluorescence in the ventricle of + */218A*, we further bred this strain with transgenic lines *Tg(cmlc2:mCherry)* and *Tg(flk:mCherry)*, in which the myocardium and endocardium are labeled, respectively. We also labeled the epicardium with an anti-pan-cytokeratin (Pck) antibody [19]. We observed that the GFP signal in + */218A* larval hearts specifically overlapped with the myocardial marker, but not the endocardial or epicardial markers (Fig. 1B), which indicated that GFP expression in + */218A* ventricle is specific to CMs.

The transposon integration site in *218A* was analyzed via Southern blotting and inverse PCR, and was mapped in intron 3 of the *csrp3* genomic locus. This was predicted to result in truncated *csrp3* transcription due to the SV40-poly A element (Fig. 1C). RT-PCR and sequencing analysis further confirmed the existence of hybrid transcripts consisting of truncated csrp3 fused with the insertion sequence (Fig. 1D) and the absence of wild-type full-length *csrp3* transcripts in *218A* homozygous fish, while + /*218A* heterozygous fish possessed both wild-type and hybrid *csrp3* transcripts (Fig. 1E). Zebrafish csrp3 encodes a polypeptide of 193 amino acids that shares highly conserved sequence and functional domains with its human and mouse orthologs (Figure S2). Whole-mount in situ hybridization (WISH) analysis revealed that endogenous *csrp3* was also specifically expressed in the zebrafish ventricle during early larval development (Fig. 1G), in line with the expression pattern of GFP in + */218A*. Further investigation showed that wild-type *csrp3* transcription was abolished in *218A* homozygous fish, as validated by the lack of wild-type Csrp3 protein (Fig. 1F, G). These data suggested that *gSAIzGFFM218A* harbors an insertion in the *csrp3* genomic locus and that heterozygous + /*218A* can be used as a reliable reporter line of endogenous *csrp3* expression, while homozygous *218A* serves as a *csrp3* mutant line.

### Csrp3 is involved in trabeculation, and its expression is regulated by hemodynamics and Notch signaling during heart development

**Fig. 2:**
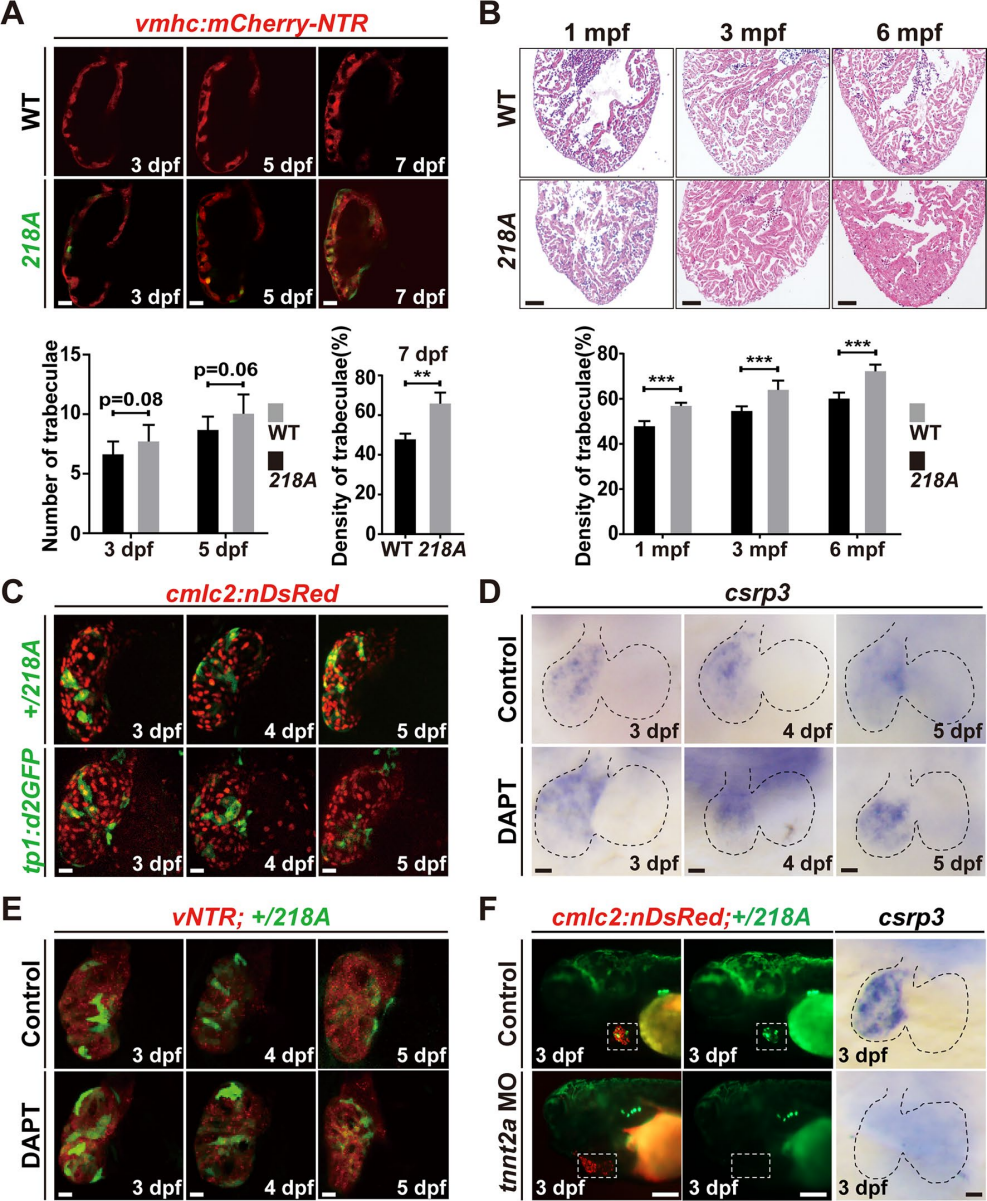
Csrp3 is involved in trabeculation, and its expression is regulated by hemodynamics and Notch signaling during heart development. **A** Upper: visualization of trabeculae using *Tg(vmhc:mCherry-NTR)* displayed increased trabeculation in *218A* larval hearts. Scale bars, 20 μm. Bottom: Quantification of the number or density of trabeculae in wild-type and *218A* larval hearts at indicated stages. N = 9 each. Data are presented as mean ± SD, Student’s t-test, **, *p* < 0.01. **B** Upper: HE staining showed an increased myocardial density in *218A* adult hearts. Scale bars, 100 μm. Bottom: Quantification of the myocardial density in wild-type and *218A* adult hearts. N = 6 each. Data are presented as mean ± SD, Student’s t-test, ***, *p* < 0.001. **C** Comparison of the fluorescence pattern in the hearts of + /*218A* and *Tg(tp1:d2GFP)* larvae. Scale bars, 20 μm. **D**, **E** WISH and confocal images showed that inhibition of Notch signaling with DATP treatment enhanced both endogenous *csrp3* expression in wild-type hearts (**D**) and GFP fluorescence in + */218A* hearts (**E**). Scale bars, 20 μm. **F** Confocal and WISH images showed that reducing blood flow via *tnnt2a* MO injection markedly suppresses both GFP fluorescence in + */218A* hearts and endogenous *csrp3* expression in wild-type hearts. Scale bars, 20 μm

The *218A* larvae were visually indistinguishable in gross morphology from wild-type and heterozygous larvae and grew normally to adulthood with comparable fertility (Figure S1A). Given the cardiac expression of endogenous *csrp3* and the + /*218A* GFP fluorescence, we further focused on the heart development of *218A* fish. They displayed regular heart shape, similar heart rates and ventricular size compared to wild types (Figure S1B–H). Although Csrp3 is suggested to be involved in myofibril organization and the maintenance of the contractile apparatus [20], we did not observe apparent structural abnormalities by anti-Tnnt2 (CT3) immunostaining (Figure S1D). Instead, we observed excessive trabeculation in *218A* larval and adult hearts. A slightly greater number of trabeculae appeared in the ventricle wall at 3 and 5 dpf when trabeculation was initiated. Later, a higher myofibril density of the trabeculae region was observed at 7 dpf and in adult hearts of *218A* fish than in those of wild-type fish at the corresponding stages (Fig. 2A, B), indicating that Csrp3 deficiency caused excessive growth of trabeculae during heart development. Meanwhile, we also observed a marked reduction of N-cadherin (CDH2) on the surface of outer layer CMs in the *218A* larval hearts (Figure S3), implying the impairment of cell–cell adhesion which may facilitate the delamination and growth of trabeculae.

Previous studies have elucidated the critical role of Notch signaling in trabeculation [21, 22]. Interestingly, the dynamic mosaic pattern of GFP in + */218A* hearts resembled the fluorescence pattern in the heart of *Tg(tp1:d2GFP)* (Fig. 2C), a reporter line expressing destabilized GFP in Notch-activated cells [21]. To determine whether Notch signaling acts as a regulator of *csrp3* expression, we inhibited Notch signaling with the specific inhibitor DAPT from 60 to 72 h post-fertilization (hpf). The results showed that inhibition of Notch signaling enhanced both GFP fluorescence in + */218A* hearts and endogenous *csrp3* expression in wild-type hearts during larval stages (Fig. 2D, E).

Blood flow also plays a crucial role during trabeculation [23, 24]. We next examined the impact on *csrp3* expression when hemodynamics was altered via *tnnt2a* morpholino (MO) injection at one-cell stage. Compared with control MO, injection of *tnnt2a* MO completely abolished the expression of both GFP in + */218A* hearts and endogenous *csrp3* in wild-type hearts (Fig. 2F). Since *tnnt2a* knockdown disturbed zebrafish cardiac development, we sought to further validate this observation by treating the larvae with two anesthetics, tricaine and 2,3-butanedione monoxime (BDM), for a short period to temporarily reduce blood flow. Consistently, we observed that both the GFP signal in + */218A* hearts and endogenous *csrp3* expression in wild-type hearts were markedly suppressed (Figure S4). Overall, these results suggested that Csrp3 is involved in zebrafish cardiac trabeculation, and that blood flow and Notch signaling play opposing roles in the regulation of *csrp3* expression during heart development.

### *csrp3* expression increases in response to zebrafish heart injury

**Fig. 3:**
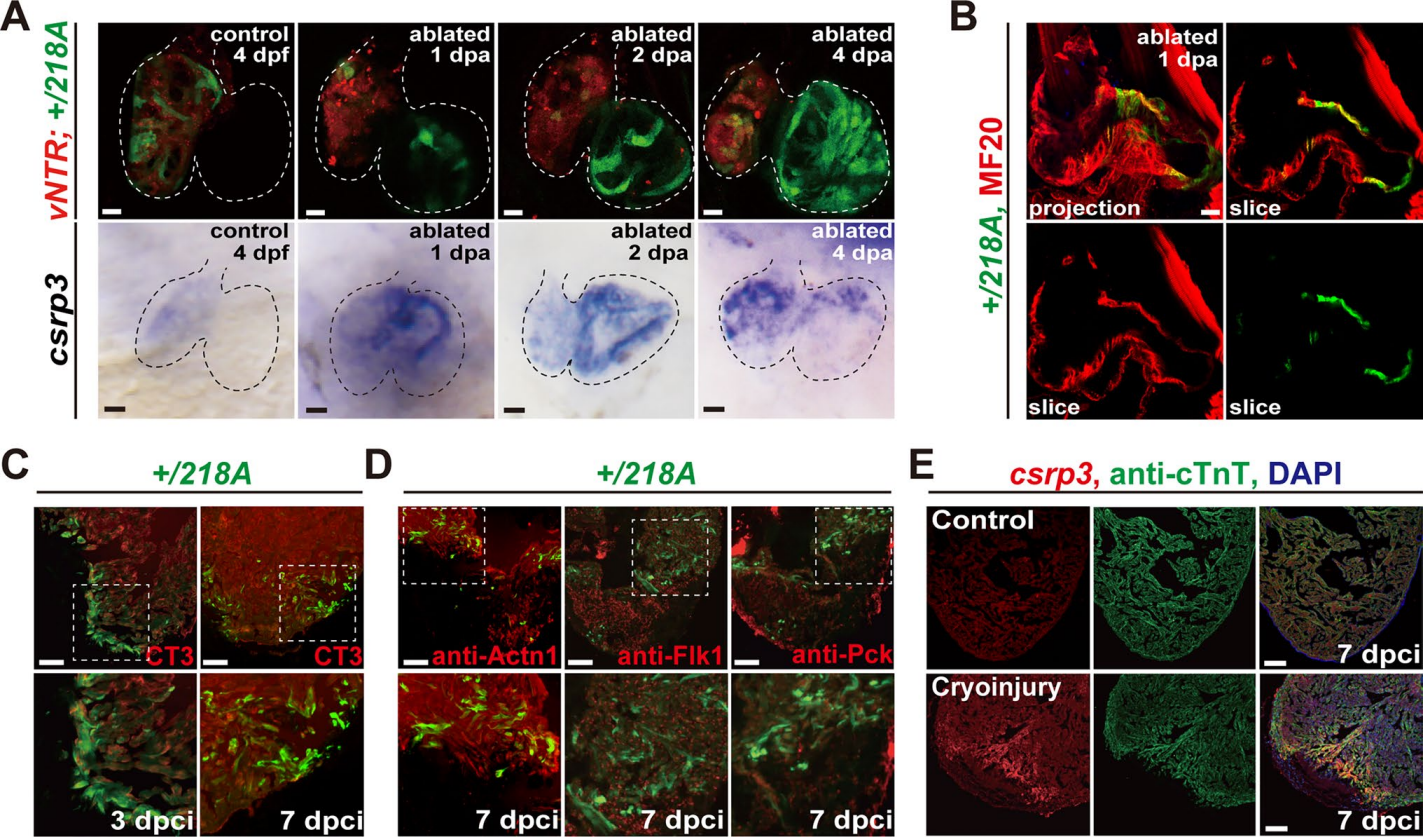
*csrp3* expression increases in response to zebrafish heart injury. **A** The temporal expression profiles of GFP in + */218A* hearts (upper) and endogenous *csrp3* in wild-type hearts (bottom) after ventricle ablation. Scale bars, 20 μm. **B** Immunostaining showed the colocalization of GFP fluorescence with MF20-marked myocardium in + /*218A* larval hearts after ventricle ablation. Scale bar, 20 μm. **C** Confocal images illuminated a significant increase of GFP fluorescence in TnnT2(CT3)-marked myocardial cells at the border zone of the cryoinjured + */218A* adult ventricle. Areas of dashed boxes are magnified. Scale bars, 100 μm. **D** Immunostaining revealed that GFP fluorescence of + */218A* was specifically expressed in myocardial (Actn1^+^) cells, while absent in the endocardial (Flk^+^) and epicardial (Pck^+^) cells. Areas of dashed boxes are magnified. Scale bars, 100 μm. **E** Fluorescence in situ hybridization (FISH) analysis corroborated a robust upregulation of *csrp3* expression at the border zone of cryoinjured ventricle, compared to the faint and dispersive signal in the uninjured adult ventricle. Scale bars, 100 μm

To investigate whether Csrp3 also plays a role in heart regeneration, we first examined its spatiotemporal expression profiles in various zebrafish heart injury models. We mated + */218A* with *Tg(vmhc:mCherry-NTR)*, a well-established ventricle ablation line [11], and treated with metronidazole (MTZ) to ablate ventricular CMs at 3 dpf. Instead of restricted ventricle expression in uninjured control hearts, the GFP signal was strongly upregulated in the atrium of ablated hearts from 1 day post-ablation (dpa) (Fig. 3A, upper panel). The GFP signal gradually expanded over the atrium and later increased in the ventricle from 2 to 4 dpa, and was restricted in the CMs in both chambers, as it overlapped with immunostaining for the myocardial marker MHC (MF20 antibody) (Fig. 3B). WISH analysis revealed a more dynamic endogenous *csrp3* expression in wild-type ablated hearts during larval ventricle regeneration. The staining was first dramatically increased in the atrium at 1 dpa, and gradually extended to the ventricle at later stages (Fig. 3A, lower panel).

Additionally, we performed cryoinjury on the ventricle of adult + */218A* fish at 6 months of age. We observed a significant increase in GFP fluorescence at the border zone of the injured area at 3 days post-cryoinjury (dpci), which became more extensive at 7 dpci (Fig. 3C). Fluorescence in situ hybridization (FISH) analysis corroborated a robust upregulation of *csrp3* expression in the injury border zone of the ventricle, compared to the faint and dispersed *csrp3* expression in the uninjured adult hearts (Fig. 3E). Colocalization of GFP with the myocardial marker Actn1 in the injured + */218A* hearts further affirmed the myocardium-specific expression of *csrp3*, while GFP was absent in the endocardial and epicardial cells, marked by Flk and Pck, respectively (Fig. 3D). These findings indicated that *csrp3* expression increased in response to heart injury in zebrafish, and may play a role in heart regeneration.

### Csrp3 deficiency impedes zebrafish heart regeneration by reducing CM proliferation and enhancing apoptosis

**Fig. 4:**
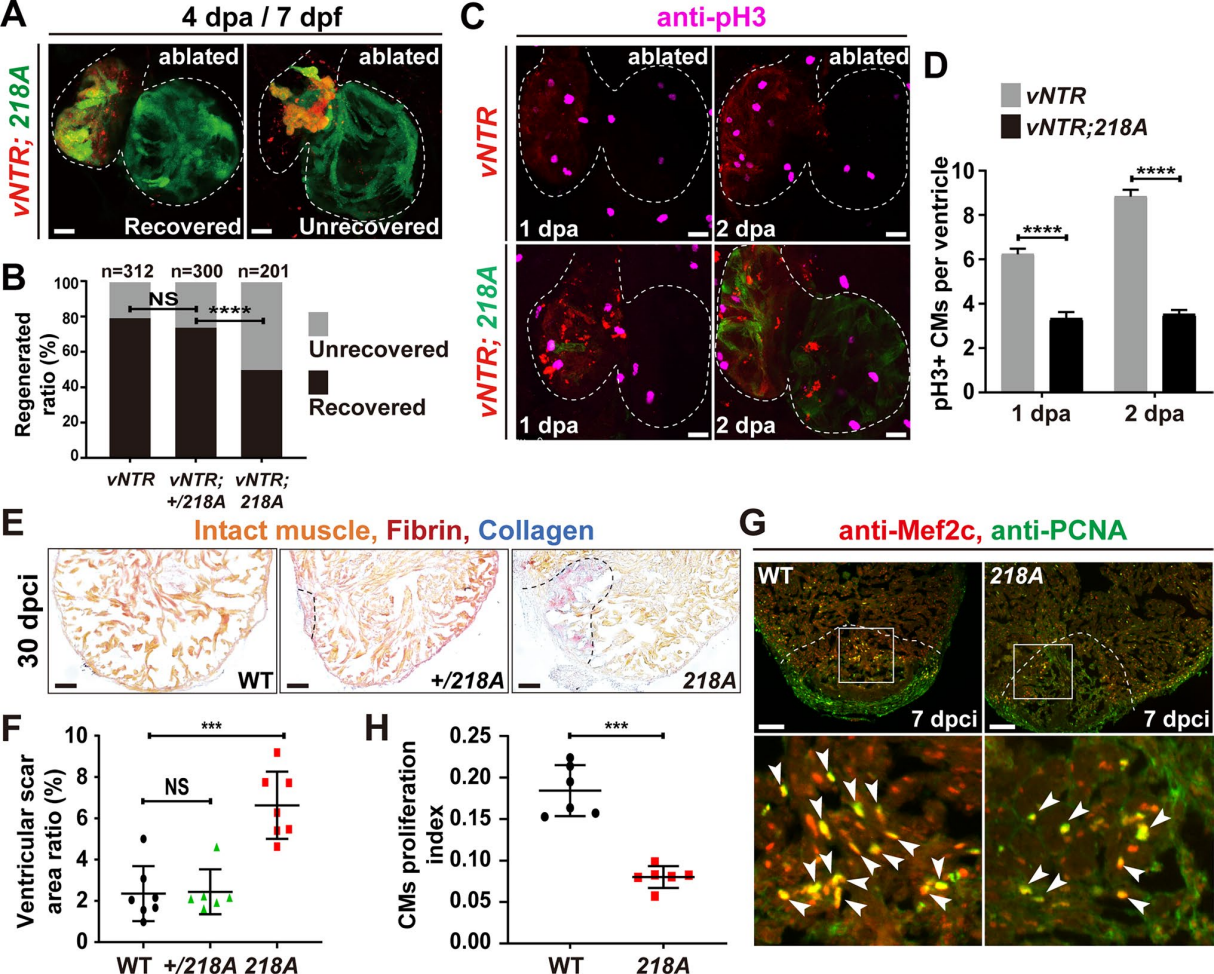
Csrp3 deficiency reduces CM proliferation and impedes zebrafish heart regeneration. **A** Representative images of the recovered/ unrecovered heart of *218A* mutant larvae at 4 dpa/7 dpf. Scale bars, 20 μm. **B** Quantification of the regeneration ratio of ablated wild-type, + */218A*, and *218A* hearts at 4 dpa/7 dpf. The numbers of larvae analyzed are indicated. Binomial test, ****P < 0.0001, NS, non-significant. **C** Immunostaining of the mitotic marker phospho-histone H3 (pH3) revealed a significant decrease of proliferating CMs in *218A* larval hearts at indicated stages. Scale bars, 20 μm. **D** Quantification of the number of pH3^+^ CMs in ablated wild-type and *218A* hearts. N = 18 each. Data are presented as mean ± SD, Student’s t-test, ****, *p* < 0.0001. **E** Representative AFOG staining of cryoinjured ventricles from wild-type, + */218A*, and *218A* fish at 30 dpci. Scale bars, 100 μm. **F** Quantification of ventricular scar area ratio in wild-type, + */218A*, and *218A* fish at 30 dpci. N = 7, 6, 7, respectively. Student’s t-test, ***, *p* < 0.001, NS, non-significant. **G** Representative confocal images of cryoinjured ventricle sections from wild-type and *218A* adult fish at 7 dpci stained with anti-PCNA (green) and anti-Mef2c (red) antibodies. Box areas are amplified. Arrowheads indicate proliferating CMs. Scale bars, 100 μm. **H** Quantification of CM proliferation index at border zone and injury site of ventricle sections at 7 dpci. N = 6 each. Student’s t-test, ***, *p* < 0.001

After ventricle ablation the injured hearts regenerated through CM proliferation, dedifferentiation/transdifferentiation and migration, and full contractile function was restored at 4 dpa [13, 25]. However, we found that *csrp3* deficiency notably impeded ventricle regeneration. Quantification of the heart regeneration ratio (number of recovered larvae over total injured larvae) revealed that the percentage of recovered larvae was significantly reduced to 49.7% (N = 201) in the ablated *218A* group compared to 79.7% (N = 312) in the ablated wild-type group and 74.3% (N = 300) in the ablated + /*218A* group (Fig. 4A, B). We then assessed CM proliferation in *218A* larvae using immunostaining of the mitotic marker phospho-histone H3 (pH3) and observed a significant decrease in the number of pH3-positive CMs in *218A* ventricles compared to wild-type ventricles. The number of pH3-positive CMs was reduced from 6.2 ± 0.3 and 8.8 ± 0.3 per ventricle in wild type to 3.3 ± 0.3 and 3.5 ± 0.2 per ventricle in the *218A* mutant at 1 and 2 dpa, respectively (Fig. 4C, D).

Furthermore, hearts of *218A* adult fish at 6 mpf were subjected to ventricular cryoinjury and then assayed for fibrin and fibrotic scar tissue using acid fuchsin-orange G (AFOG) staining at 30 dpci. We observed that *218A* hearts retained large unrecovered wounds with prominent fibrin or collagen deposits in comparison to minimal scar patches in injured wild-type and + */218A* hearts accompanied by substantial new cardiac myofiber formation (Fig. 4E, F). We next evaluated injury-induced CM proliferation by conducting immunostaining of the DNA replication marker PCNA and CM marker Mef2c at 7 dpci. Notably, the number of PCNA-positive CMs was markedly decreased in *218A* mutant hearts. Quantification revealed an approximately 56% (0.18 ± 0.03 to 0.09 ± 0.01) decrease in the CM proliferation index (PCNA^+^Mef2C^+^ cells/Mef2C^+^ cells) in *218A* hearts compared to wild-type hearts (Fig. 4G, H).

**Fig. 5:**
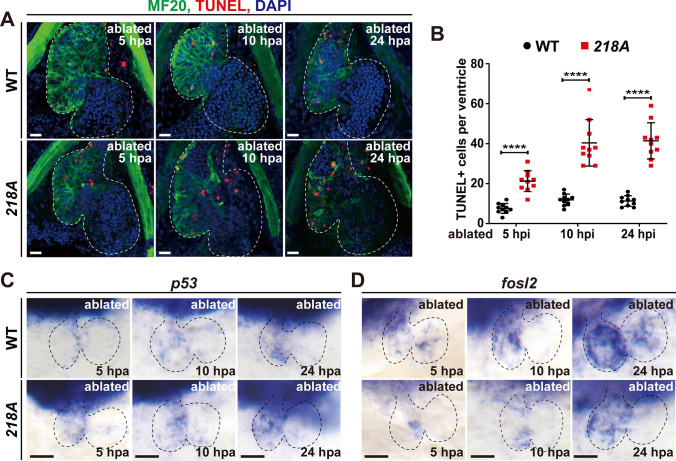
Csrp3 deficiency results in elevated CM apoptosis. **A** TUNEL assay showed significantly increased apoptosis signals in the *218A* larval hearts at the early stages after ablation. Scale bars, 20 μm. **B** Quantification of TUNEL-positive CMs in the ablated ventricle of wild-type and *218A* larval at indicated stages. N = 10 each. Student’s t-test, ****, *p* < 0.0001. **C**, **D** WISH showed the upregulation of apoptosis-related gene *tp53* and the downregulation of antiapoptotic gene *fosl2* in *218A* larval hearts. Scale bars, 50 μm

In addition, the apoptosis of CMs after ventricle ablation was also assessed using TUNEL assays, which revealed that apoptotic signals were profoundly increased in *218A* larval hearts, from 7.6 ± 2.3, 11.9 ± 2.7 and 11.3 ± 2.5 per wild-type ventricle to 21.2 ± 5.5, 40.4 ± 12.9 and 41.4 ± 9.9 per *218A* larval ventricle at 5, 10 and 24 h post-ablation (hpa), respectively (Fig. 5A, B). WISH also revealed the upregulation of the apoptosis-related gene *tp53* as well as the downregulation of antiapoptotic gene *fosl2* (Fig. 5C, D). In general, these results elucidated the crucial role of Csrp3 in zebrafish heart regeneration, indicating that Csrp3 deficiency impeded ventricle regeneration by reducing CM proliferation and enhancing apoptosis.

### Csrp3 deficiency impairs injury-induced CM dedifferentiation and sarcomere reassembly

**Fig. 6:**
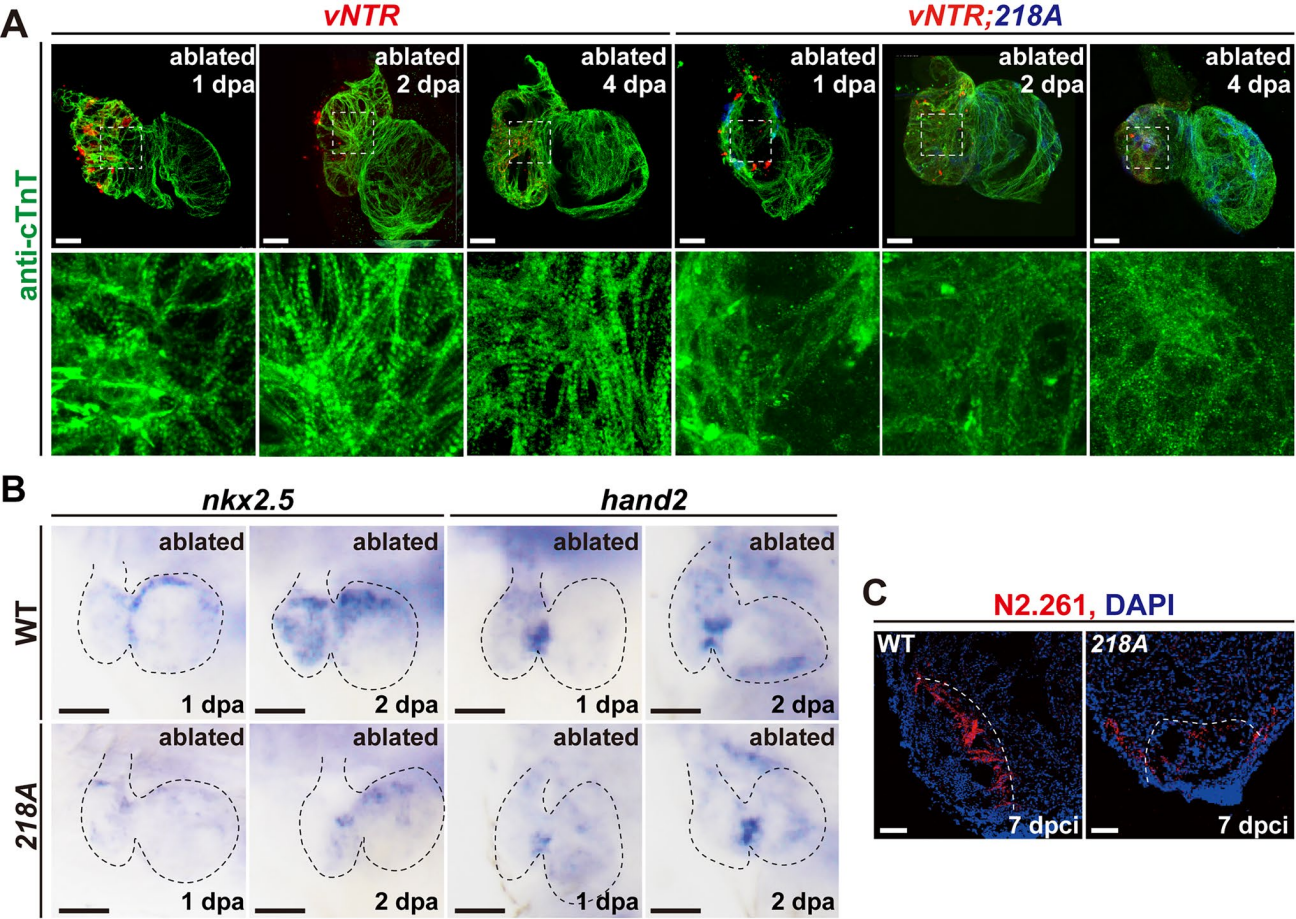
Csrp3 deficiency impairs injury-induced CM dedifferentiation and sarcomere reassembly. **A** Immunostaining of cTnT indicated that sarcomere disassembly of *218A* CMs was not blocked but instead exacerbated during regeneration while sarcomere reassembly was hindered. Scale bars, 20 μm. **B** WISH showed reduced expression of cardiogenic factors *nkx2.5* and *hand2* in ablated *218A* larval hearts during regeneration. Scale bars, 50 μm. **C** Immunostaining showed reduced embryonic Myh7 signal in the border zone of injured *218A* adult ventricle at 7 dpci. Scale bars, 100 μm

Sarcomere disassembly and re-expression of key early cardiac transcriptional regulators are essential processes for the dedifferentiation and subsequent proliferation of preexisting CMs during zebrafish heart regeneration [19, 26]. Immunostaining of cardiac troponin T (cTnT) showed that sarcomere disassembly of *218A* CMs was not blocked at 1 dpa but was instead exacerbated during regeneration, characterized by disarrayed, and even collapsed arrangements. This in turn hindered sarcomere reassembly at later stages of regeneration (Fig. 6A). Meanwhile, we further examined the expression of several cardiogenic factors. In line with the effect of *csrp3* deficiency on CM proliferation during regeneration, *csrp3* deficiency sharply reduced the expression of *nkx2.5* and *hand2* in ablated *218A* larval hearts (Fig. 6B). The level of embryonic CM marker Myh7 (stained with the N2.261 antibody) was also decreased in the border zone of injured *218A* adult ventricle compared to that in the wild-type hearts (Fig. 6C). These results suggested that Csrp3 may have an important role in CM dedifferentiation and is indispensable for sarcomere reassembly during zebrafish heart regeneration.

Previous studies demonstrated that Csrp3 played an essential role in the CM stretch sensor machinery [2], and was required for mechanical stability in skeletal muscles [27]. We recently showed mechanotransduction is critical for zebrafish heart regeneration [28]. Thus, we also assessed the expression of several genes involved in mechanotransduction process in ablated *218A* larval hearts. We observed a significant downregulation of *peizo1* and a slight upregulation of *integrin-linked kinase* (*ilk*) in the *218A* ablated hearts at 2 dpa compared to wild-type ablated hearts (Figure S5), indicating a disturbance of mechanotransduction. These data implied that Csrp3 might also play a crucial role in mechanotransduction to facilitate zebrafish heart regeneration, while the exact mechanisms warrant further exploration.

### Multiple signaling pathways regulate *csrp3* expression in response to zebrafish larval heart ablation

**Fig. 7:**
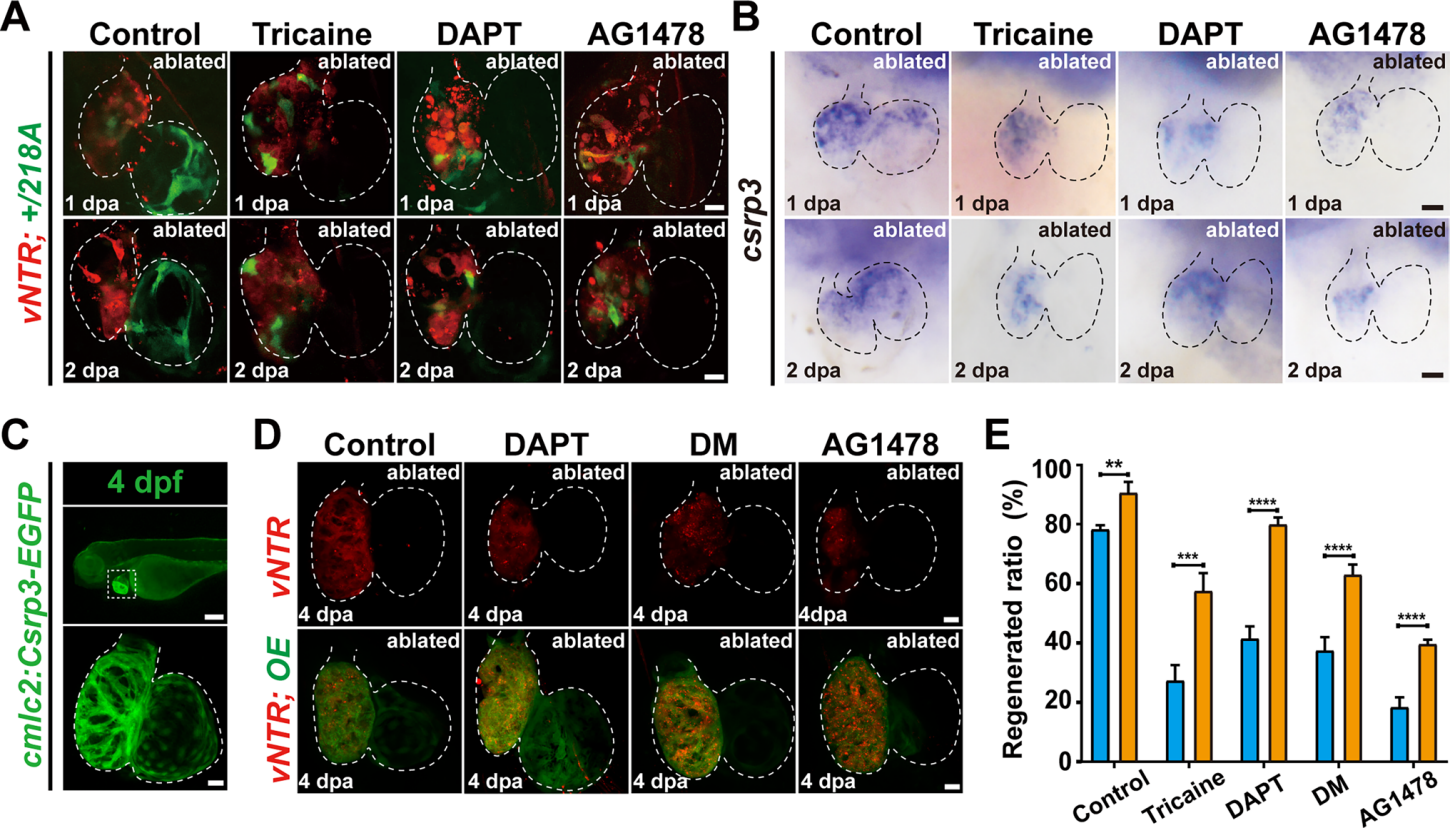
*csrp3* overexpression relieves the inhibitory effects of multiple signaling blockage on zebrafish heart regeneration. **A**, **B** Comparative analysis of the expression changes of GFP in ablated + */218A* hearts and endogenous *csrp3* in ablated wild-type hearts after inhibiting blood flow (tricaine), Notch (DAPT), and ErbB (AG1478) signaling. Scale bars, 20 μm. **C** Fluorescence pattern of *Tg(cmcl2:Csrp3-EGFP)* at 4 dpf. Scale bars, 20 μm. **D** Confocal images showed that Csrp3 overexpression (OE) ameliorated the impairment of heart regeneration resulting from inhibiting blood flow (tricaine), Notch (DAPT), and ErbB (AG1478) signaling. Scale bars, 20 μm. **E** Quantification of the regeneration ratio of ablated wild-type and Csrp3 overexpressed larvae after treatment with indicated inhibitors at 4 dpa/7 dpf. N = 481, 548, 487, 411, 689, 524, 312, 328, respectively. Data are presented as mean ± SD, Student’s t-test, **P < 0. 01, ***P < 0.001, ****P < 0.0001

Studies have shown that multiple signaling pathways are activated to orchestrate the regeneration of injured zebrafish hearts [29–31], including hemodynamics, Notch signaling, ErbB signaling, BMP signaling, etc. Pharmacological experiments were conducted to explore whether *csrp3* expression responded to the suppression of these crucial signaling pathways which resulted in a failure of zebrafish heart regeneration (Figure S6A). Blood flow reduction by tricaine or inhibition of endocardial Notch activation by DAPT markedly blocked the appearance of GFP signal in the atrium of ablated + */218A* hearts (Fig. 7A). WISH also revealed dismissed endogenous *csrp3* expression in the atrium and reduced expression in the ventricle of ablated wild-type hearts treated with tricaine or DAPT (Fig. 7B). Pharmacological inhibition of ErbB signaling with AG1478 treatment induced a similar effect on + */218A* GFP fluorescence and *csrp3* expression following heart ablation (Fig. 7A, B**)**, while inhibitors for Wnt signaling (cardiomogen-1), BMP signaling (dorsomorphin, LDN193189), and mTOR signaling (rapamycin) showed negligible impacts on GFP intensity in ablated + */218A* hearts and endogenous *csrp3* expression in ablated wild-type hearts (Figure S6B, C).

### *csrp3* overexpression relieves the inhibitory effects of multiple signaling blockage on zebrafish heart regeneration

To investigate the potential contribution of Csrp3 gain of function to zebrafish heart regeneration, we generated a stable transgenic line *Tg(cmlc2:Csrp3-EGFP)*, that specifically overexpresses Csrp3 in CMs. *Tg(cmlc2:Csrp3-EGFP)* larvae exhibited normal gross morphology and intact heart structure with striated myofibers (Fig. 7C). After ventricle ablation, Csrp3 overexpression promoted CM proliferation (Figure S7) and further increased the heart regeneration ratio at 4 dpa from 78.5% (N = 481) to 90.7% (N = 548) (Fig. 7D, E). Intriguingly, Csrp3 overexpression partially ameliorated the impairment of heart regeneration resulting from the suppression of blood flow, Notch signaling, and ErbB signaling (Fig. 7D, E). The heart regeneration ratio at 4 dpa increased from 27.5% (N = 487), 41.6% (N = 689) and 18.7% (N = 312) in tricaine-treated, DAPT-treated and AG1478-treated ablated wild-type larvae to 57.7% (N = 411), 80.1% (N = 524) and 39.8% (N = 328) in ablated *Tg(cmlc2: Csrp3-EGFP)* larvae with corresponding inhibitor treatments. Csrp3 overexpression also reversed the altered expressions of mechanotransduction-related genes in *218A* ablated hearts (Figure S5).

## Discussion

In the current study, through analysis of the novel gene-trap line *gSAIzGFFM218A*, we revealed the dynamic patterns and essential roles of *csrp3* in zebrafish heart development and regeneration. The *218A* fish survive to adulthood with normal gross morphology, while the CSRP3/MLP-null mice exhibit severe heart failure leading to mortality [32]. This discrepancy may be partially attributed to the residual functions of truncated Csrp3 peptides in *218A* fish. Besides, as a critical scaffold protein for maintaining cardiac muscle structure and function, the dysfunction of Csrp3 would likely have a more severe impact on mice hearts than on zebrafish hearts since mice have much higher heart rates and stronger cardiac contractions compared to zebrafish, which indicates that the mouse heart is subjected to greater mechanical forces and requires higher rigidity. Previously we showed by WISH that *csrp3* expression was restricted in larval ventricle with a mosaic pattern [27]. Colocalization analysis with markers of different cardiac layers in the current study revealed that the GFP fluorescence of heterozygous + */218A* larvae was restricted to the ventricular CMs, resembling the fluorescence pattern in the Notch signaling reporter line *Tg(tp1:d2GFP)* [21]. Interestingly, both GFP fluorescence in + */218A* fish and endogenous *csrp3* expression in wild-type fish changed in response to alterations in hemodynamic force and Notch signaling activity, two well-known factors regulating cardiac trabeculation [22, 33, 34]. Indeed, homozygous *218A* fish with Csrp3 deficiency caused excessive growth of trabeculae during heart development, as evidenced by the increased myofibril density in the trabecular area, which may be partially attributed to the reduction and mislocalization of certain junctional proteins. We did not observe such phenotype in our previously reported *csrp3* knockout fish [27], which may be due to the genetic compensation response triggered in many CRISPR knockout [35–37]. However, abnormal trabeculation was found in human patients with left ventricular non-compaction (LVNC) in which *CSRP3* defect is believed to contribute to the etiology [38]. Taken together, our results indicated that *csrp3* is involved in trabeculation during heart development, yet the exact molecular mechanism and its relationship with Notch signaling warrants further investigation.

The role of *csrp3* in zebrafish heart regeneration has not been examined before. We observed a significant reduction of regeneration ratio after larval ventricle ablation or impeded regeneration with elevated scar tissue after adult cryoinjury in *218A* fish compared to wild type. Csrp3 deficiency also blocked CM proliferation in both injury models. We further generated a myocardial-specific Csrp3 overexpression line and found that OE-Csrp3 increased CM proliferation and regeneration ratio after larval ventricle ablation. Previous studies have proven that autophagy is involved in zebrafish heart regeneration [39–41]. Through *CSRP3* silence, autophagy impairment also resulted in increased apoptosis in both myoblasts and myotubes [42, 43]. We indeed observed elevated apoptosis after heart injury in *218A* fish by TUNEL staining. WISH results showed increased expression of apoptosis-related gene *tp53* and reduced expression of antiapoptotic gene *fosl2* [44]. CMs spared from apoptosis will undergo dedifferentiation-proliferation-redifferentiation to replace the lost myocardium [45–47]. Disassembly and reassembly of sarcomere is a critical step of this process [26, 48]. We found in *218A* fish, sarcomere disassembly was significantly aggravated at 1 dpa, which in turn hindered the sarcomere reassembly at later stages of regeneration. We also found altered expressions of mechanotransduction-related genes in ablated *218A* hearts. Overall, our results revealed the essential roles of *csrp3* in zebrafish heart regeneration.

Meanwhile, *csrp3* displayed a dynamic expression pattern during heart regeneration. GFP signal in + */218A* larvae was strongly upregulated in the atrium of ablated hearts, gradually expanded over the atrium, and later increased in the ventricle. Multiple signaling pathways orchestrated heart regeneration [30], and some of them were shown to regulate *csrp3* expression in the current study. We found that *csrp3* activation in the atrium was eliminated with the treatment of inhibitors to block hemodynamics, Notch, and ErbB signaling pathways, but remained no change with the treatment of inhibitors for Wnt, BMP, and mTOR signaling. Whether other players in mechanotransduction also regulate *csrp3* activation remains to be explored. Furthermore, OE-Csrp3 ameliorated the impairment of heart regeneration resulting from the blockage of blood flow, Notch, and ErbB signaling pathways by pharmacological inhibitors. We previously showed *lepb*-linked enhancer sequence (*LEN*) was activated in the endocardium of ablated hearts and its activity was also regulated by hemodynamic forces and Notch signaling [31]. With the restricted myocardial expression during heart regeneration and dynamic response to certain signaling pathways, + */218A* would be a valuable addition to our toolsets for dissecting the epistasis and cross-talking of signaling networks during heart regeneration.

### Supplementary Information

Below is the link to the electronic supplementary material.

Supplementary file1 (PDF 2075 KB)

## Data Availability

All data supporting the findings of this study are available within the article and the Supplementary Materials.
