# Correction: A novel gene-trap line reveals the dynamic patterns and essential roles of *cysteine and glycine-rich protein 3* in zebrafish heart development and regeneration

**DOI:** 10.1007/s00018-024-05334-9

**Published:** 2024-09-10

**Authors:** Shuzhang Liang, Yating Zhou, Yue Chang, Jiayi Li, Min Zhang, Peng Gao, Qi Li, Hong Yu, Koichi Kawakami, Jinmin Ma, Ruilin Zhang

**Affiliations:** 1 TaiKang Medical School (School of Basic Medical Sciences), Wuhan University, Wuhan, 430071 China; 2 School of Life Sciences, Fudan University, Shanghai, 200433 China; 3 Shanghai Key Laboratory of Regulatory Biology, Institute of Molecular Medicine, School of Life Sciences, East China Normal University, Shanghai, 200241 China; 4 Shanghai Pediatric Congenital Heart Disease Institute and Pediatric Translational Medicine Institute, Shanghai Children’s Medical Center, Shanghai Jiao Tong University School of Medicine, Shanghai, 200127 China; 5 Institute of Myocardial Injury and Repair, Wuhan University, Wuhan, 430071 China; 6 Hubei Provincial Key Laboratory of Developmentally Originated Disease, Wuhan, 430071 China; 7 Laboratory of Molecular and Developmental Biology, National Institute of Genetics, Mishima, Shizuoka 411-8540 Japan; 8 Department of Genetics, Graduate University for Advanced Studies (SOKENDAI), Mishima, Shizuoka 411-8540 Japan; 9 Medical Frontier Innovation Research Center, The First Hospital of Lanzhou University, The First Clinical Medical College of Lanzhou University, Lanzhou, 730000 China

**Correction to: Cellular and Molecular Life Sciences (2024) 81:158**

10.1007/s00018-024-05189-0

In the published article Fig. 7 contain mislabeling and miss bar symbols. Fig. S6 contain mislabeling. The corrected figures are as follow.

**Fig. 7:**
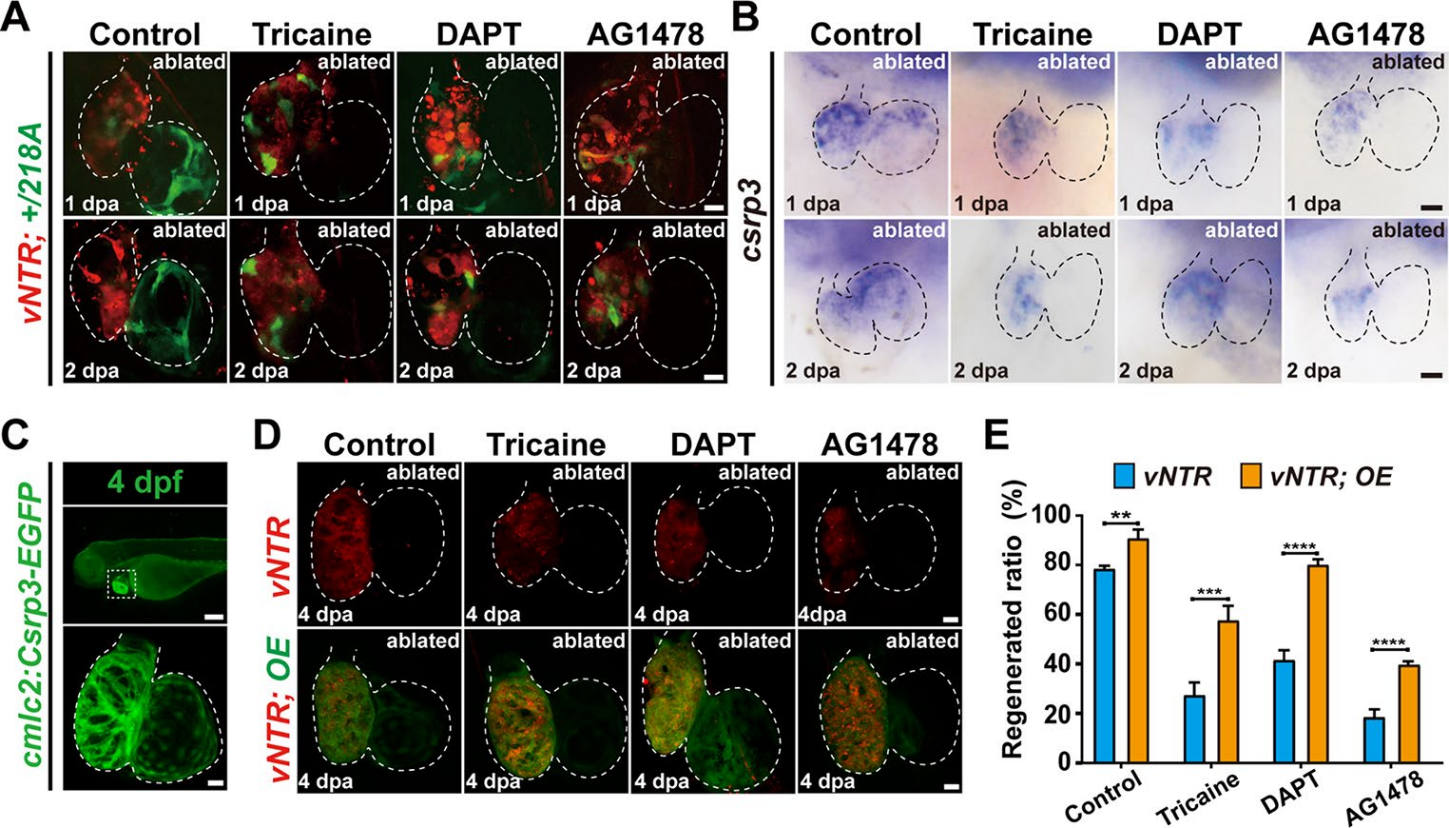
*csrp3* overexpression relieves the inhibitory effects of multiple signaling blockage on zebrafish heart regeneration. **A**,** B** Comparative analysis of the expression changes of GFP in ablated *+/218A* hearts and endogenous *csrp3* in ablated wild-type hearts after inhibiting blood flow (tricaine), Notch (DAPT), and ErbB (AG1478) signaling. Scale bars, 20 μm. **C** Fluorescence pattern of *Tg(cmcl2:Csrp3-EGFP)* at 4 dpf. Scale bars, 20 μm. **D** Confocal images showed that Csrp3 overexpression (OE) ameliorated the impairment of heart regeneration resulting from inhibiting blood flow (tricaine), Notch (DAPT), and ErbB (AG1478) signaling. Scale bars, 20 μm. **E** Quantification of the regeneration ratio of ablated wild-type and Csrp3 overexpressed larvae after treatment with indicated inhibitors at 4 dpa/7 dpf. *N* = 481, 548, 487, 411, 689, 524, 312, 328, respectively. Data are presented as mean ± SD, Student’s t-test, **, *p* < 0. 01, ***, *P* < 0.001, ****, *P* < 0.0001

**Fig. S6:**
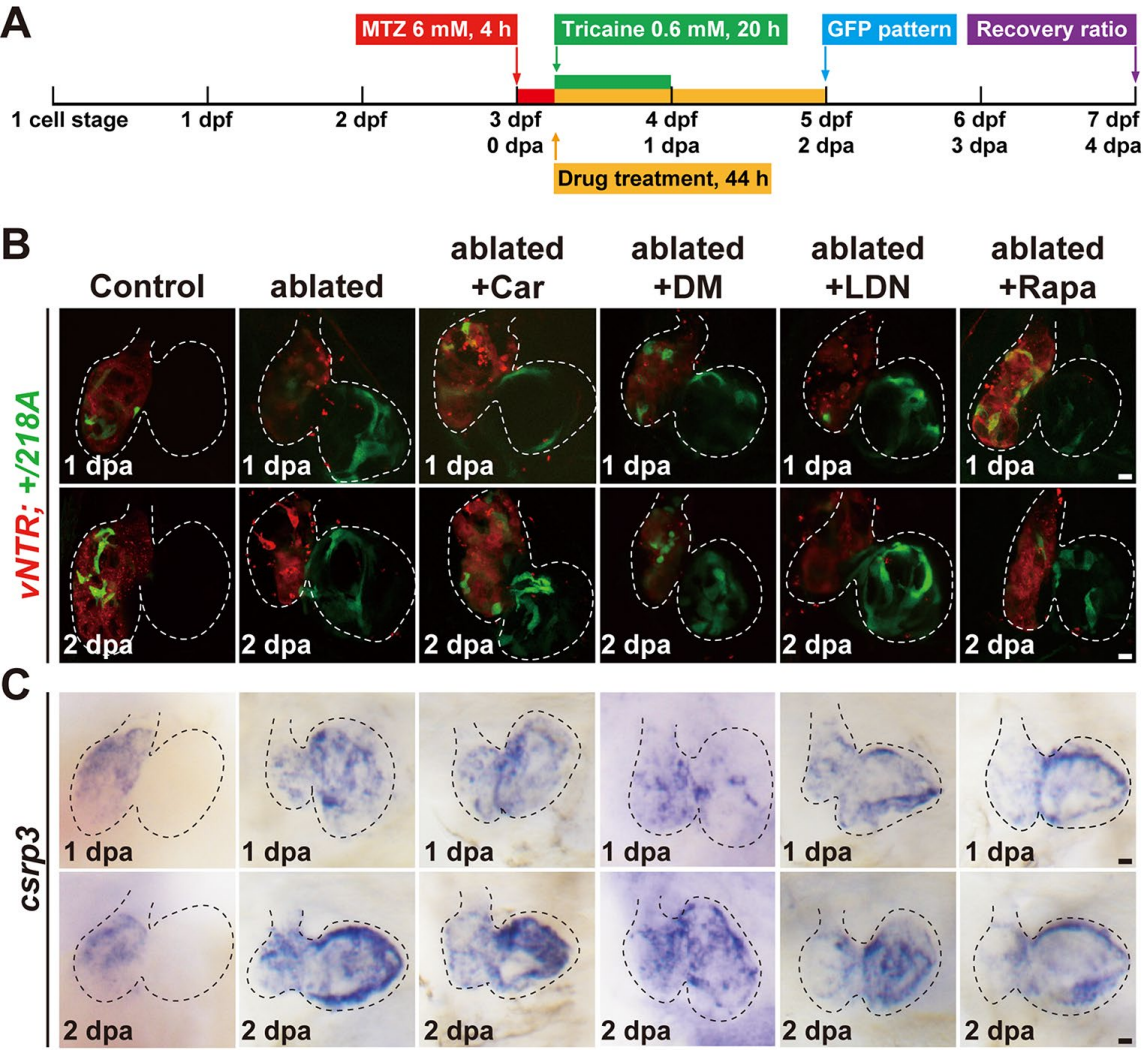
The effects of pharmacological blockage of multiple signaling pathways on *csrp3* expression during zebrafish heart regeneration. **A** Schematic timeline diagram of the pharmacological experiments. **B**,** C** Fluorescence images and WISH showed the expression changes of GFP in *+/218A* hearts and endogenous *csrp3* in wild-type hearts treated with Wnt inhibitor cardiomogen-1 (Car), BMP inhibitors dorsomorphin (DM) and LDN193189 (LDN), and mTOR inhibitor rapamycin (Rapa). Scale bars, 20 μm

The original article has been updated.

## Electronic supplementary material

Below is the link to the electronic supplementary material.

Supplementary Material 1

