# Supplementary material for: A novel gene-trap line reveals the dynamic patterns and essential roles of cysteine and glycine-rich protein 3 in zebrafish heart development and regeneration
Source: Cell Mol Life Sci. 2024 Mar 31;81(1):158. doi: 10.1007/s00018-024-05189-0 (PMC10982097; doi:10.1007/s00018-024-05189-0)
Supplement: Supplementary file 1 [file 18_2024_5189_MOESM1_ESM.pdf]

Supplementary Materials

Supplementary Figure 1. The gross and heart morphology of *218A* homozygous fish at larval and adult stages

Supplementary Figure 2. Zebrafish Csrp3 shares highly conserved protein sequence and functional domains with its human and mouse orthologs

Supplementary Figure 3. Decreased expression and altered distribution of N-Cadherin in the hearts of *218A* homozygous larvae

Supplementary Figure 4. Reducing blood flow suppresses *csrp3* expression in zebrafish larval hearts

Supplementary Figure 5. Mechanotransduction-related gene expression in response to the alteration of *csrp3* level during zebrafish heart regeneration

Supplementary Figure 6. The effects of pharmacological blockage of multiple signaling pathways on *csrp3* expression during zebrafish heart regeneration

Supplementary Figure 7. Csrp3 overexpression promotes CM proliferation during zebrafish heart regeneration

Supplementary Table 1. List of primers used in this study

Supplementary Table 2. List of antibodies used in this study

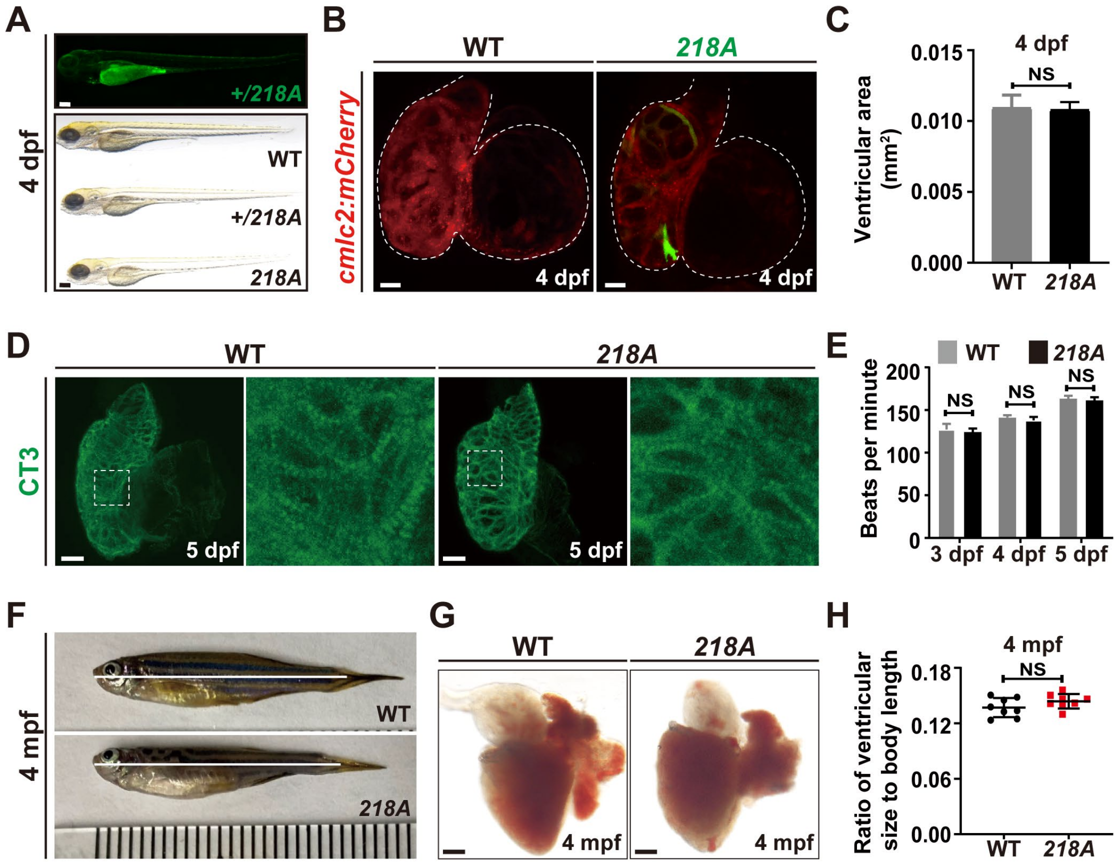

Supplementary Figure 1. The gross and heart morphology of *218A* homozygous fish at larval and adult stages

(A) Gross morphology of wild-type, *+/218A* and *218A* larvae at 4 dpf. Scale bars, 200 µm.

(B-E) *218A* larvae exhibited normal heart shape (B), ventricular size, N=10 each (C), myofibril organization (D), and regular heart rates, N=11 each (E). Scale bars, 20 µm. Data are presented as mean ± SD, Student's t-test, NS, non-significant.

(F, G) Comparison of body and heart morphology of wild-type and *218A* adult fish at 4 mpf. Scale bars, 200 µm.

(H) The ratio of ventricle size to body length of *218A* adult fish was comparable to that of wild types. N=8 each. Student's t-test, NS, non-significant.

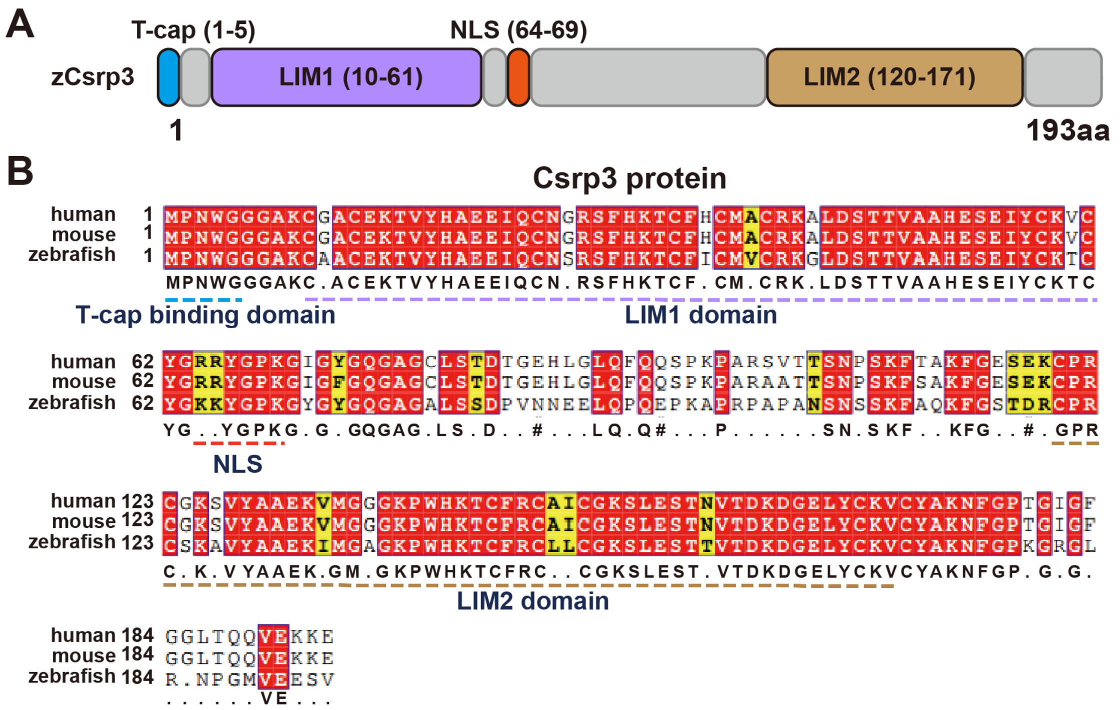

Supplementary Figure 2. Zebrafish Csrp3 shares highly conserved protein sequence and functional domains with its human and mouse orthologs

(A) Schematic diagram illustrating the domain organization of zebrafish Csrp3.

(B) Sequence alignment of human, mouse, and zebrafish Csrp3 proteins. Red shading indicates identical amino acids; yellow shading indicates conserved amino acids. Dashed lines indicate the corresponding functional domain.

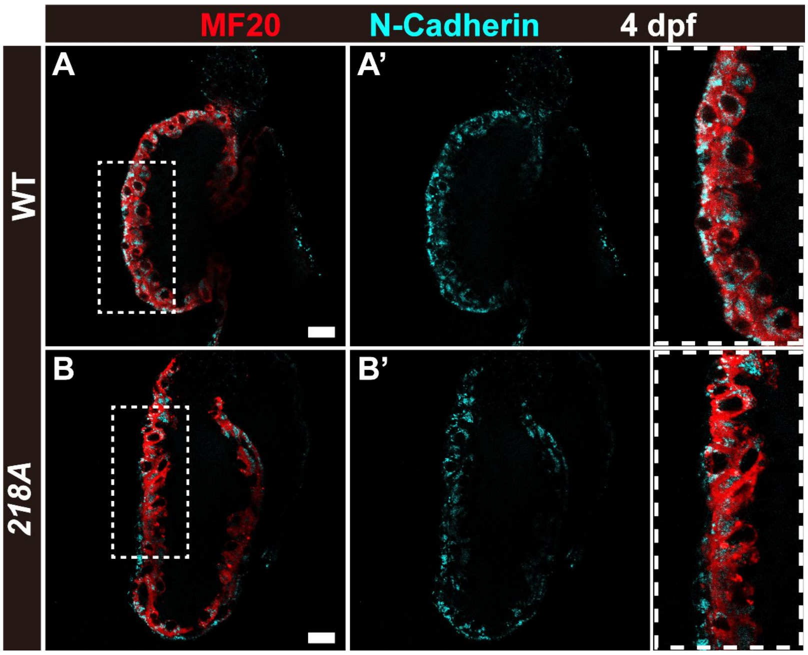

Supplementary Figure 3. Decreased expression and altered distribution of N-Cadherin in the hearts of *218A* homozygous larvae

Immunostaining of the junctional protein N-Cadherin in wild-type (A, A') and *218A* (B, B') larval hearts at 4 dpf. Areas of dashed boxes are magnified. Scale bars, 20 µm.

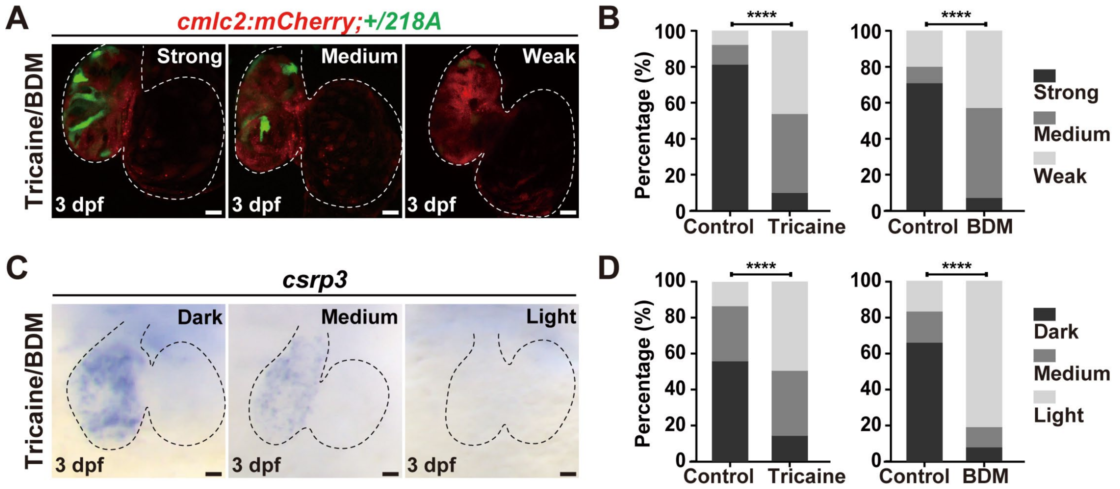

Supplementary Figure 4. Reducing blood flow suppresses *csrp3* expression in zebrafish larval hearts

(A) Representative fluorescence images of different classes of GFP intensity in the *+/218A* larval hearts treated with tricaine or BDM at 3 dpf. Scale bars, 20 µm.

(B) Quantification of the percentages of different classes of GFP intensity in the *+/218A* larval hearts treated with or without tricaine/BDM. N=83, 69, 118, 60 respectively. Chi-square test, ****P<0.0001.

(C) Representative WISH images of different levels of endogenous *csrp3* expression in wild-type larval hearts treated with tricaine or BDM at 3 dpf. Scale bars, 20 µm.

(D) Quantification of the percentages of different levels of *csrp3* expression in wild-type larval hearts treated with or without tricaine/BDM. N=98, 95, 68, 80 respectively. Chi-square test, ****P<0.0001.

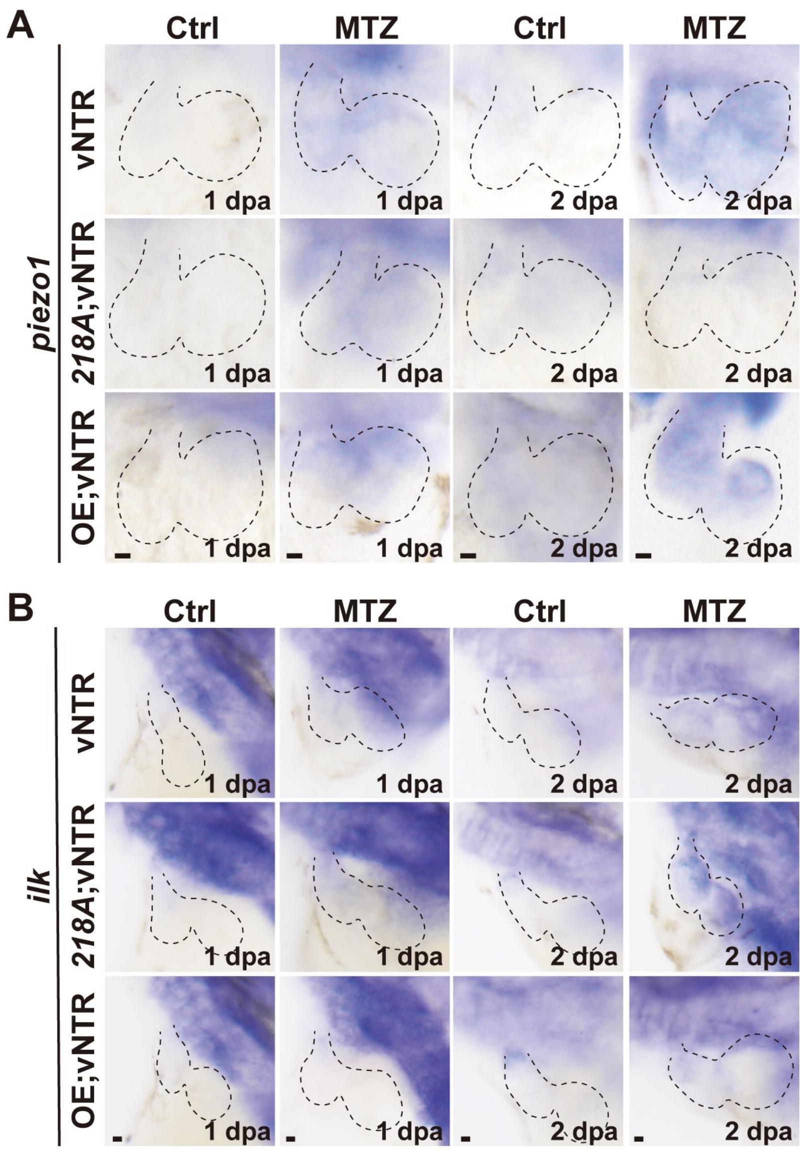

Supplementary Figure 5. Mechanotransduction-related gene expression in response to the alteration of *csrp3* level during zebrafish heart regeneration

Whole-mount *in situ* hybridization of *peizo1* (A), and *ilk* (B). Scale bars, 20 µm.

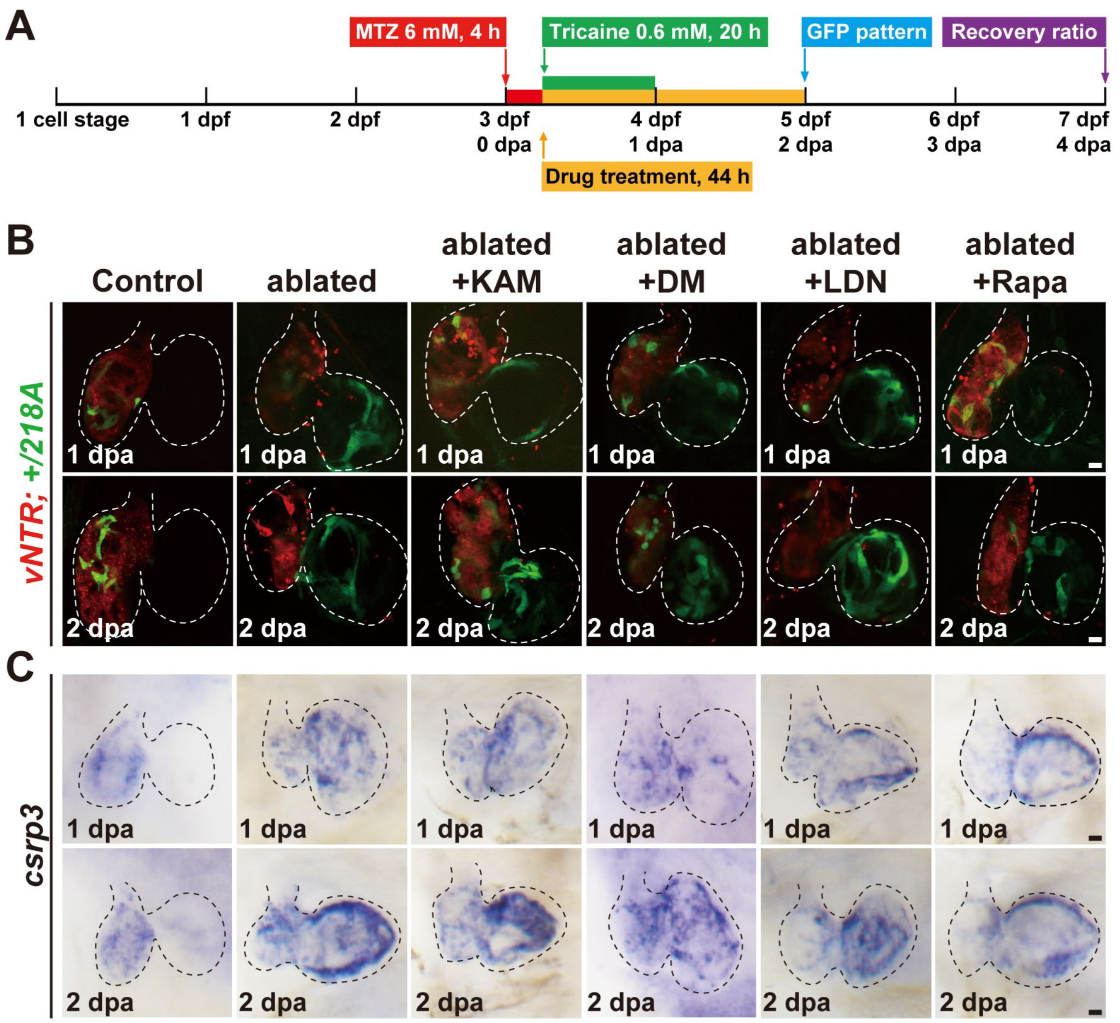

Supplementary Figure 6. The effects of pharmacological blockage of multiple signaling pathways on *csrp3* expression during zebrafish heart regeneration

(A) Schematic timeline diagram of the pharmacological experiments.

(B, C) Fluorescence images and WISH showed the expression changes of GFP in *+/218A* hearts and endogenous *csrp3* in wild-type hearts treated with Wnt inhibitor cardiomogen-1 (Car), BMP inhibitors dorsomorphin (DM) and LDN193189 (LDN), and mTOR inhibitor rapamycin (Rapa). Scale bars, 20 μm.

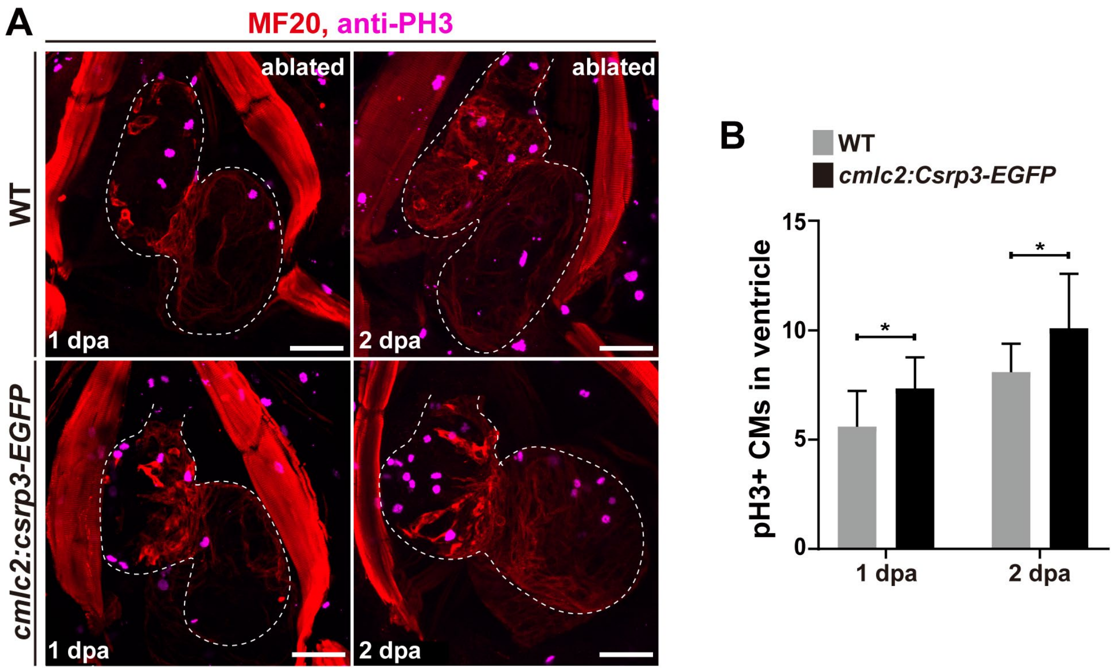

Supplementary Figure 7. Csrp3 overexpression promotes CM proliferation during zebrafish heart regeneration

(A) Immunostaining of the mitotic marker phospho-histone H3 (pH3) to assess the capacity of CM proliferation in ablated wild-type and *Tg(cmlc2: Csrp3-EGFP)* larval hearts at indicated stages. Scale bars, 20 µm.

(B) Quantification of the numbers of $pH3^+$ CMs in ablated wild-type and *Tg(cmlc2: Csrp3-EGFP)* hearts at indicated stages. N=20 each. Data are presented as mean ± SD, Student's t-test, *, $p < 0.05$.

Supplementary Table 1. List of primers used in this study

Gene	Forward primer (5'-3')	Reverse primer (5'-3')
**WISH probes**		
csrp3	ATGGTTTGTCGTAAAGGTTTGGA	CTTTAATCTCCAATGGAAAGTCGTT
tp53	TTGTCCCATATGAAGCACCA	TCAGAGTCGCTTCTTCCTTC
fosl2	TGGATCATGTACCAGGATTACACC	TGCTGGGAATGTGAGCTCTG
nkx2.5	CATACTGAACCTGGAGCAGAAT	CATCCCAGCCAAACCATATCTCA
hand2	CCCCTATTTTCATGGGTGGCT	AAGTTTGTGTCTTCGGACGGA
piezo1	GCAGTCCATCCAGCCCTTTACA	TGATTCTTCTCTCTCGTCCACT
ilk	GGGCACAGAGATTGTGGTCA	CCTCTTTGCAGGGTCTTCGT
**Genotyping primers**		
F1	AAGGTTTGGACAGCACCACA	
R1	AGGCTCTTCCCGCACAATAA	
R2	CTATCCAACTCACAACGTGGCA	

Supplementary Table 2. List of antibodies used in this study

Antibody	SOURCE	IDENTIFIER
Mouse monoclonal anti-Tnnt2 (CT3)	DSHB	Cat#AB_528495
Mouse monoclonal anti-Myh1e (MF20)	DSHB	Cat#AB_2147781
Mouse monoclonal anti-Myh7 (N2.261)	DSHB	Cat#AB_531790
Chicken monoclonal anti-GFP	Abcam	Cat#ab13970
Mouse monoclonal anti-Pck	Abcam	Cat#ab6401
Rabbit polyclonal anti-N Cadherin	Abcam	Cat# ab18203
Rabbit polyclonal anti-cTnT	Cell Signaling Technology	Cat#5593
Mouse monoclonal anti-Actn1	Sigma-Aldrich	Cat#A7732
Mouse polyclonal anti-Flk1	Sigma-Aldrich	Cat# P35918
Mouse monoclonal anti-PCNA	Sigma-Aldrich	Cat#P8825
Rabbit polyclonal anti-Mef2c	Santa Cruz Biotechnology	Cat#sc-313
Rabbit polyclonal anti-pH3 (Ser10)	Millipore	Cat#06-570
Goat polyclonal anti-Csrp3	Invitrogen	Cat#PA5-19062
Alexa Fluor 488 goat anti-chicken IgG	Invitrogen	Cat#A11039
Alexa Fluor 488 goat anti-mouse IgG	Invitrogen	Cat#A28175
Alexa Flour 555 goat anti-mouse IgG	Invitrogen	Cat#A28180
Alexa Flour 488 goat anti-rabbit IgG	Invitrogen	Cat#A11034
Alexa Flour 555 goat anti-rabbit IgG	Invitrogen	Cat#A27039
